# Towards a cardiac allocation score: a retrospective calculation for 73 patients from a German transplant center

**DOI:** 10.1186/s13019-017-0575-7

**Published:** 2017-03-07

**Authors:** Sebastian Claes, Michael Berchtold-Herz, Qian Zhou, Georg Trummer, Matthias Bock, Andreas Zirlik, Friedhelm Beyersdorf, Christoph Bode, Sebastian Grundmann

**Affiliations:** 1 Heart Center Freiburg University, Department of Cardiology and Angiology I, Faculty of Medicine, University of Freiburg, Hugstetter Strasse 55, 79106 Freiburg, Germany; 2 Heart Center Freiburg University, Department of Cardiovascular Surgery, Faculty of Medicine, University of Freiburg, Freiburg, Germany

**Keywords:** Heart transplantation, Prognosis, Heart failure

## Abstract

**Background:**

Due to a growing discrepancy between the transplant waiting list and decreasing numbers of available donor hearts, cardiac transplantation rates in Germany have been declining in the past years. Currently, patients on the waiting list are prioritized by medical urgency and waiting time and therefore a majority of all cardiac transplants is performed in very ill patients. Recently, a different allocation algorithm was proposed that included predicted post-transplant survival as a parameter for organ allocation. So far, little data exists on how such a “Cardiac Allocation Score” (CAS) relates to our current transplant patient population and on how such a change in organ allocation could change clinical practice.

**Methods:**

We calculated a theoretical retrospective Cardiac Allocation Score for 73 patients recruited and transplanted at our medium-volume center in Germany based on a hypothetical scoring algorithm recently published by Eurotransplant.

**Results:**

Overall, 37 patients (50.7%) were transplanted on high urgency status (HU), 27 (37%) were being supported by a VAD at time of transplant. 57 (78.1%) were male. We found a relatively normal distribution of the hypothetical CAS with a median of 32.91 and a mean of 31.95 +/−10.02. Overall, CAS-Scores were lower than previously described for a Eurotransplant patient cohort of high urgency patients, but there was a significant overlap in score values between patients on HU and T status. CAS-values of VAD-supported patients were lower than in patients without mechanical support. The IMPACT-score as part of the CAS was used for prediction of post-transplant survival and seems suitable to predict outcome in our patient population.

**Conclusion:**

In a retrospective analysis, the recently proposed Cardiac Allocation Score seems to show a normal distribution of priority values in our patient cohort. The IMPACT-score predicted outcome after transplantation and could serve as part of the CAS-algorithm to predict post-transplant survival in this single center real-world scenario. Implementation of the CAS could significantly change organ allocation practice, including a potential prioritization of current T-status patients over HU-status patients.

## Background

The prevalence of heart failure is steadily increasing and has reached epidemic proportions in Germany and Europe. Approximately 1–2% of all adults suffer from heart failure, with a prevalence of more than 10% in the aging population above 70 years of age [1].

In the past decades, pharmacological treatment of heart failure with a reduced ejection fraction has been a story of great success, with significant improvements of morbidity and mortality. In addition, cardiac devices and surgical and interventional techniques now offer a wide selection of therapeutic options for patients with heart failure and are well documented to improve symptoms and/or prognosis. Overall, currently available therapies have resulted in a reduction of hospitalization events of 30–50% in comparison to the era before the introduction of ACE-inhibitors and beta-blockers [1], the current cornerstones of heart failure therapy.

Still, the outcome of patients with advanced heart failure in New York Heart Association Class III-IV remains poor. The 5-year mortality of patients hospitalized for heart failure is still approximately 50%, worse than many cancers [2]. For these patients with advanced stages of the disease and persistent symptoms under optimal therapy, orthotopic heart transplantation serves as an important treatment option to improve quality of life and prognosis. However, this treatment is limited by the availability of donor organs. In Germany, the mismatch between patients on the waiting list and available donor organs is growing and following the public discussion of recent physician misconduct in organ allocation for liver transplantation, donor numbers have come to a historic low (http://www.dso.de/dso-pressemitteilungen/einzelansicht/article/zahl-der-organspender-in-2013-weiter-stark-gesunken.html). Therefore, the allocation of the few available organs to the large number of patients in demand is a growing medical and ethical dilemma.

In the Eurotransplant region, organ allocation is prioritized by waiting time and medical urgency, where patients can be assigned a high-urgency (HU) status if they fulfill specific criteria of disease severity. While initially introduced as a rare exception to bypass the regular waiting list to allow transplantation within a period of days to a few weeks, it has now become the rule. Due to the growing number of patients on HU-status and the decline in organ donation, currently more than 80% of cardiac transplantation are performed on HU-status [3], resulting in a predominant organ allocation to the very sick patients, which intrinsically have a worse outcome also after transplantation.

Several approaches have been proposed to account for this ethical dilemma, including the prioritization of registered donors as potential recipients [4] or a change in the rules for consent to organ donation [5]. Recently, a novel scoring system for prioritization was proposed by a consortium of large European transplant centers and Eurotransplant that takes into account both waiting list mortality as well as expected post-transplant prognosis. In analogy to the established scheme for lung transplantation (the Lung Allocation Score), this novel (and so far theoretic) system was termed “Cardiac Allocation Score” (CAS) and tested on a Eurotransplant cohort of 448 patients for whom high urgency status had been applied for [6]. However, baseline characteristics of listed patients and outcome differs to a certain degree between centers and so far no published data exists on how such novel scoring system would relate to a single center transplant population. In addition, currently no published data exist on CAS-values outside the high-urgency population. Here, we therefore performed a retrospective calculation of the CAS for a cohort of 73 patients transplanted in our medium-volume transplant center in Southern Germany, including patient on all Eurotransplant urgency levels, to assess the distribution of the score and the predictive value of the CAS for our patient population.

## Methods

### Study population

From the 300 patients that underwent cardiac transplantation at the University Heart Center Freiburg-Bad Krozingen between 1994 and 2013, we selected a cohort that included all patients that were recruited for heart-only transplantation and that underwent transplantation and long-term follow up in Freiburg. For this analysis, we excluded pediatric patients aged <18 years as well as patients that were recruited and followed up outside of our department or in associated hospitals. These selections yield a study population of 92 patients, with a complete dataset enabling CAS-calculation for 73 patients. We included patients on all urgency levels. We did not analyze patients that were listed for heart transplantation but died on the waiting list.

### Pre transplant mortality model

We used the Seattle Heart Failure Model (SHFM) to retrospectively calculate the theoretical waiting list mortality for our patients, as this prediction model was recently shown to have superior predictive value in comparison to other prediction models [6]. This mortality model includes hemodynamic parameters as well as functional status and data on medication and devices [7]. As our analysis was limited to patients that survived to transplantation, our retrospective calculation cannot be regarded as a validation of the score for our cohort, but was performed to allow the calculation of the CAS as described below.

### Prediction of post-transplant mortality

We retrospectively validated two previously published mortality prediction models on our patient cohort: The Index for Mortality Prediction After Cardiac Transplantation (IMPACT) includes 12 recipient specific variables (age greater than 60, bilirubin, creatinine clearance, dialysis between listing and transplant, female sex, heart failure aetiology, infection, IABP, mechanical ventilation prior to transplant, race, temporary circulatory support, ventricular assist device) that are weighted and eventually yield a theoretical score range between 0 and 50 points. The original study design of the Impact-score excluded patients with a total artificial heart. Each value stands for a defined risk of one year mortality [8]. For our data set we separated our patient cohort in a low (0–8 points) and a high risk (>8 pts.) group.

As a second mortality model, the CARRS score was calculated for all patients. The name of this score is an acronym of the five included variables (cerebral vascular accident, albumin, re-HTx, renal dysfunction and prior sternotomies). Patients can be stratified in a high and a low risk group (0–2 pts. and >3 pts.) with a maximum of 9 points [9]. For the IMPACT-score, we chose a value of 8 points as a cut-off, as this value identifies a population with a high 1-year mortality of >15%, which we defined as high risk.

### Cardiac allocation score

The hypothetical Cardiac Allocation Score (CAS) was calculated based on the combination of the SHFM for theoretical mortality on the waiting list and the IMPACT score for post-transplant mortality prediction as recently described by Smits and coworkers [6]. Eventually, the CAS is a combination of these two scores that is adjusted to yield a maximum value of 100. Higher values identify patients with a high predicted mortality on the waiting list, but only if their predicted survival probability after cardiac transplantation is also high. Thus, the score prioritizes patients with the most benefit with regards to life time gained by cardiac transplantation.

## Results

### Demographics of the study cohort

We analysed a data set of 92 patients. Seventy-three (79.3%) patients were male with a mean age of 50.7 years. Nineteen (20.7%) were female (52.5 years). For CAS-calculation 19 patients were excluded because they were supported by a total artificial heart (which prohibits calculation of the IMPACT-score) or because data of essential variables could not be retrieved. Thirty-eight (41.3%) were supported by a VAD at time of transplantation, 8 were on extracorporal mechanical circulatory support. Thirty-nine (42.4%) were transplanted from T status, 53 (57.6%) from U or HU status. Patient characteristics are shown in Table 1.

**Table 1:** Baseline Characteristics

Variables	
Age, years	54 (18–72; 16)
Weight, kg	78 (44–123; 20)
Body mass index, kg/m^2^	25.5 (17.2–38.8; 5)
Sex, % female	20.7
Diagnosis, %	
Ischemic	38
Idiopathic	48.9
Congenital	5.4
Other	7.6
High urgency, %	56.5
Ventricular assist device^a^, %	43.2
IABP, %	12
Mechanical support (ECMO, ECLS), %	8.7
Sodium, mmol/L	138 (129–143; 4)
Creatinine, mg/dl	1.08 (0.46–3.38; 0.5)
Creatinine Clearance, ml/min	87 (31–218; 45.5)
Bilirubin, mg/dl	0.8 (0.2–5.2; 0.9)
Total Cholesterol, mg/dl	181 (95–304; 77)
Uric acid, mg/dl	6.3 (1.5–14.4; 2.6)
Hemoglobin, g/dl	11.8 (7.8–17.7; 4.1)
LVEF, %	17.3 (5–82; 5)

Data are presented as median (minimum-maximum; interquartile range)

*IABP* intra-aortic balloon pump, *ECMO* extracorporeal membrane oxygenation, *ECLS* extracorporeal life support

^a^Four Patients with Total Artificial Heart were not included

### Post-transplant survival

**Fig. 1:**
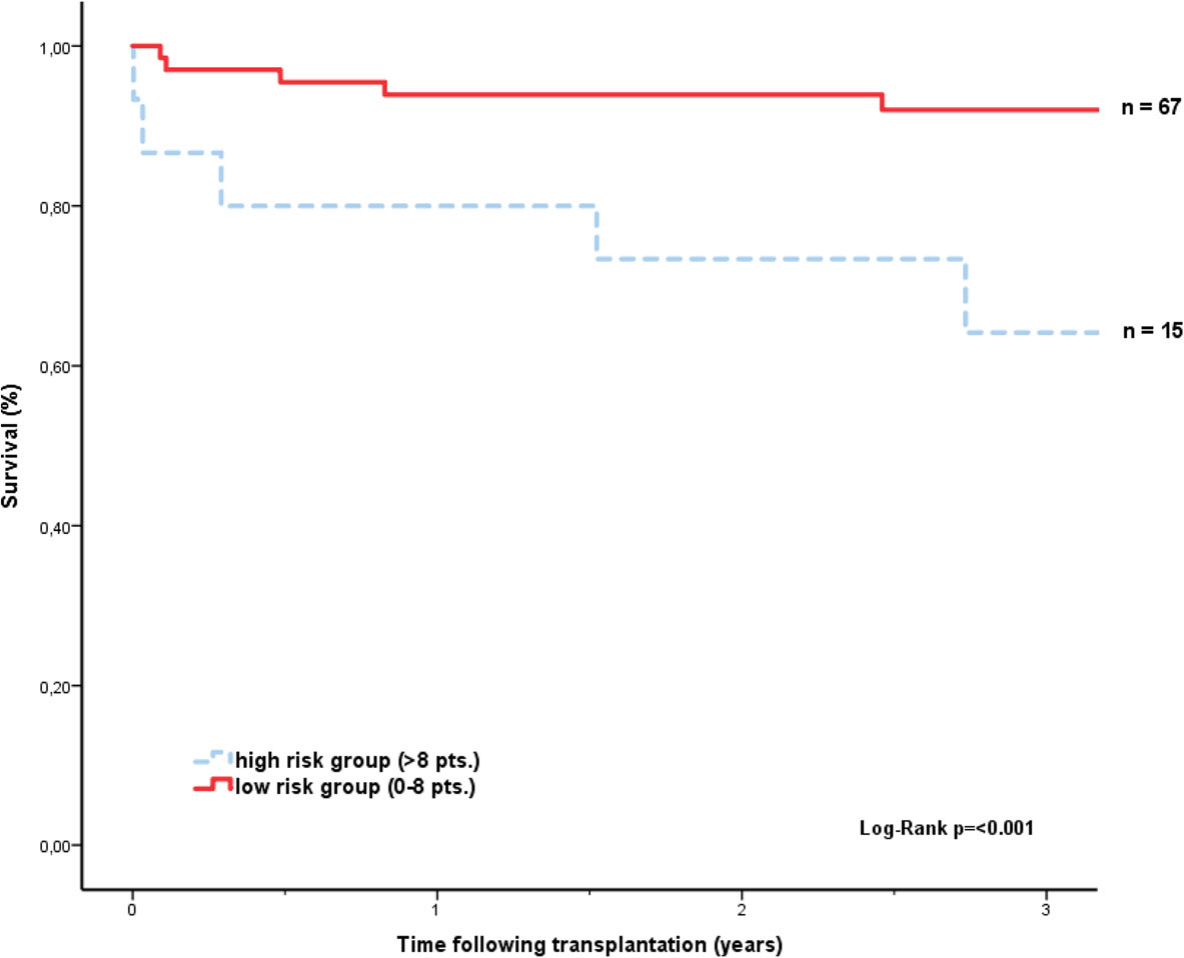
Kaplan-Meier survival curves after heart transplantation (Impact-Score)

**Fig. 2:**
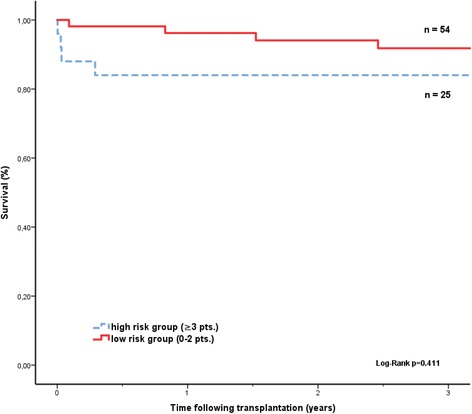
Kaplan-Meier survival curves after heart transplantation (CARRS-Score)

**Fig. 3:**
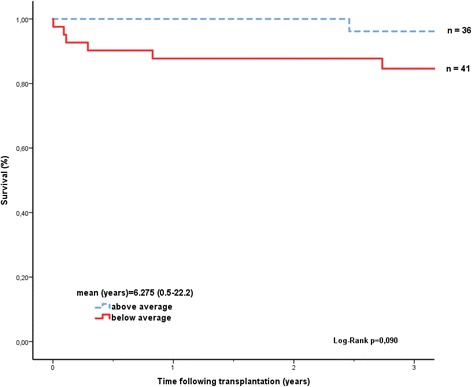
Kaplan-Meier survival curves after heart transplantation (SHFM-Score)

In our patient cohort, only the IMPACT score significantly predicted survival (Fig. 1). Patients in the higher risk stratum of the CARRS score showed a trend towards lower survival, but this did not reach statistical significance (p = 0.411, Fig. 2). Patients transplanted on HU-status had a higher IMPACT score than patients on T status at time of transplant (7.17 vs 4.11 see Table 2). The IMPACT score was predictive for survival both in the total patient cohort as well as in the VAD-supported patients. Interestingly, patients with a low waiting list mortality before transplant predicted by the SHFM also showed a trend for better survival after transplantation in our cohort (Fig. 3).

**Table 2:** Impact score factors

	Overall	T-status	HU/U status	p Value^a^
Variables				
Age, years				0.237
> 60	27	14 (35.9%)	13 (24.5%)	
≤ 60	65	25 (64.1%)	40 (75.5%)	
Bilirubin, mg/dl				0.050
0-0.99	53	28 (77.8%)	25 (49.0%)	
1-1.99	27	6 (16.7%)	21 (41.2%)	
2-3.99	6	2 (5.6%)	4 (7.8%)	
≥ 4	1	0	1 (2.0%)	
Creatinine Clearance, ml/min				0.752
≥ 50	75	30 (85.7%)	45 (88.2%)	
30-49	11	5 (14.3%)	6 (11.8%)	
< 30	0	0	0	
Dialysis between listing and transplant	5	1 (2.7%)	4 (7.5%)	0.645
Female sex	19	12 (30.8%)	7 (13.2%)	0.040
Diagnosis				0.036
Ischemic	46	16 (41.0%)	30 (56.6%)	
Idiopathic	34	20 (51.3%)	14 (26.4%)	
Congenital	5	0	5 (9.4%)	
Other	7	3 (7.7%)	4 (7.5%)	
Infection	13	1 (2.7%)	12 (22.6%)	0.026
IABP	11	1 (2.7%)	10 (18.9%)	0.024
Mechanical ventilation prior to Tx	2	0	2 (3.8%)	0.510
Circulatory support	8	0	8 (15.1%)	0.019
Ventricular assist device^d^				<0.001
No VAD	50	34 (87.2%)	16 (32.7%)	
Early Generation PF^b^	23	3 (7.7%)	20 (40.8%)	
New Generation CF^c^	3	0	3 (6.1%)	
Heartmate II	12	2 (5.1%)	10 (20.4%)	

Race is not listed because all patients were Caucasian

*IABP* Intraaortic balloon pump, *Tx* Transplantation

^a^p value based on X^2^ test or Fisher’s exact test

^b^Early Generation pulsatile flow includes Heartmate I, Thoratec (LVAD,RVAD,BVAD)), Novacor

^c^New Generation continuous flow includes Jarvik, Incor, Ventracor

^d^4 Patients with total artificial heart were not included

### Cardiac allocation score

**Fig. 4:**
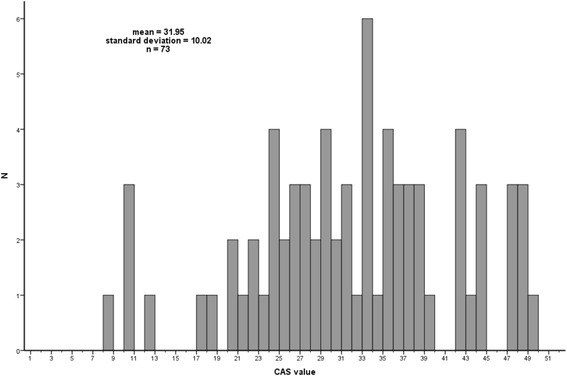
Distribution of the CAS-values across the whole cohort

**Fig. 5:**
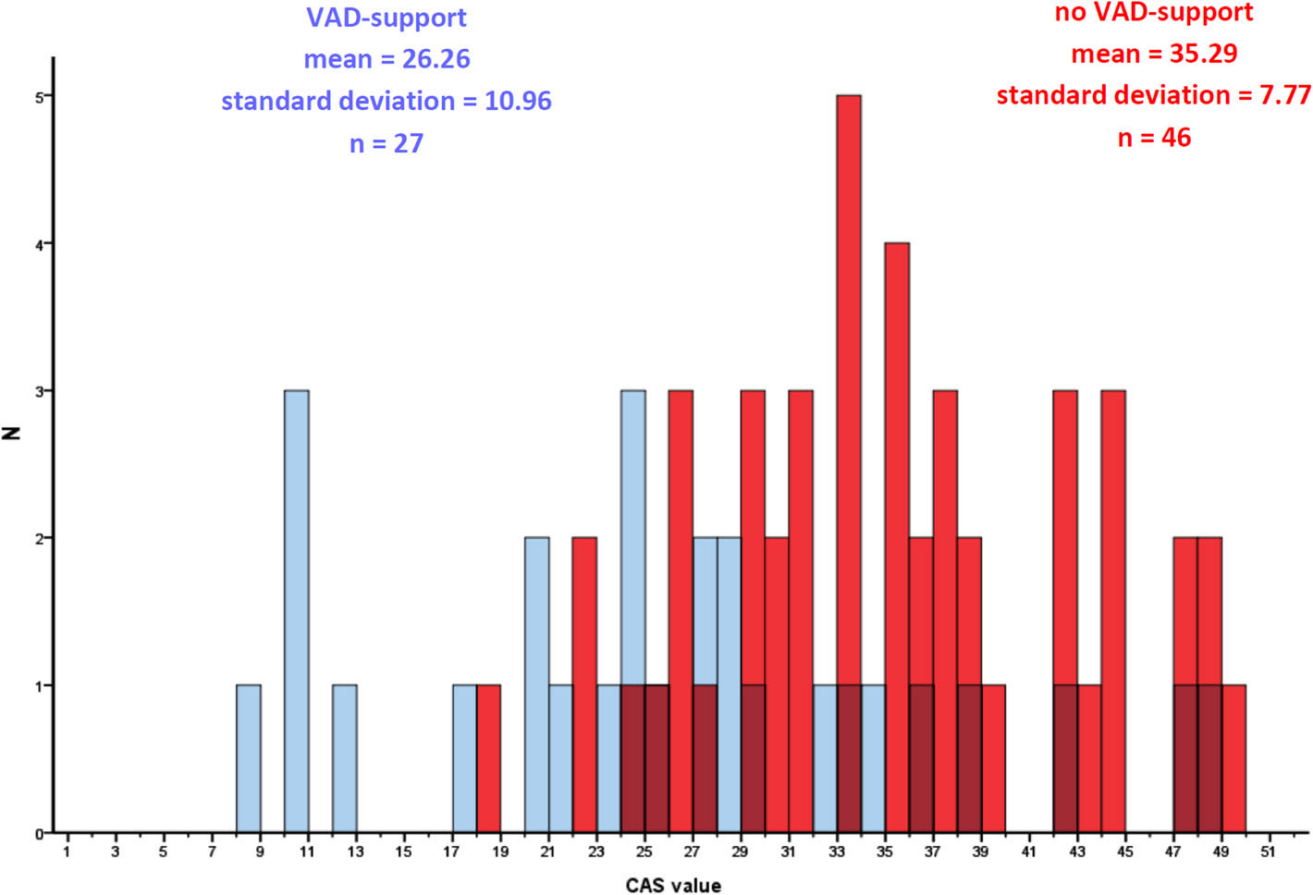
Distribution of CAS values in patients on VAD-support and without VAD

**Fig. 6:**
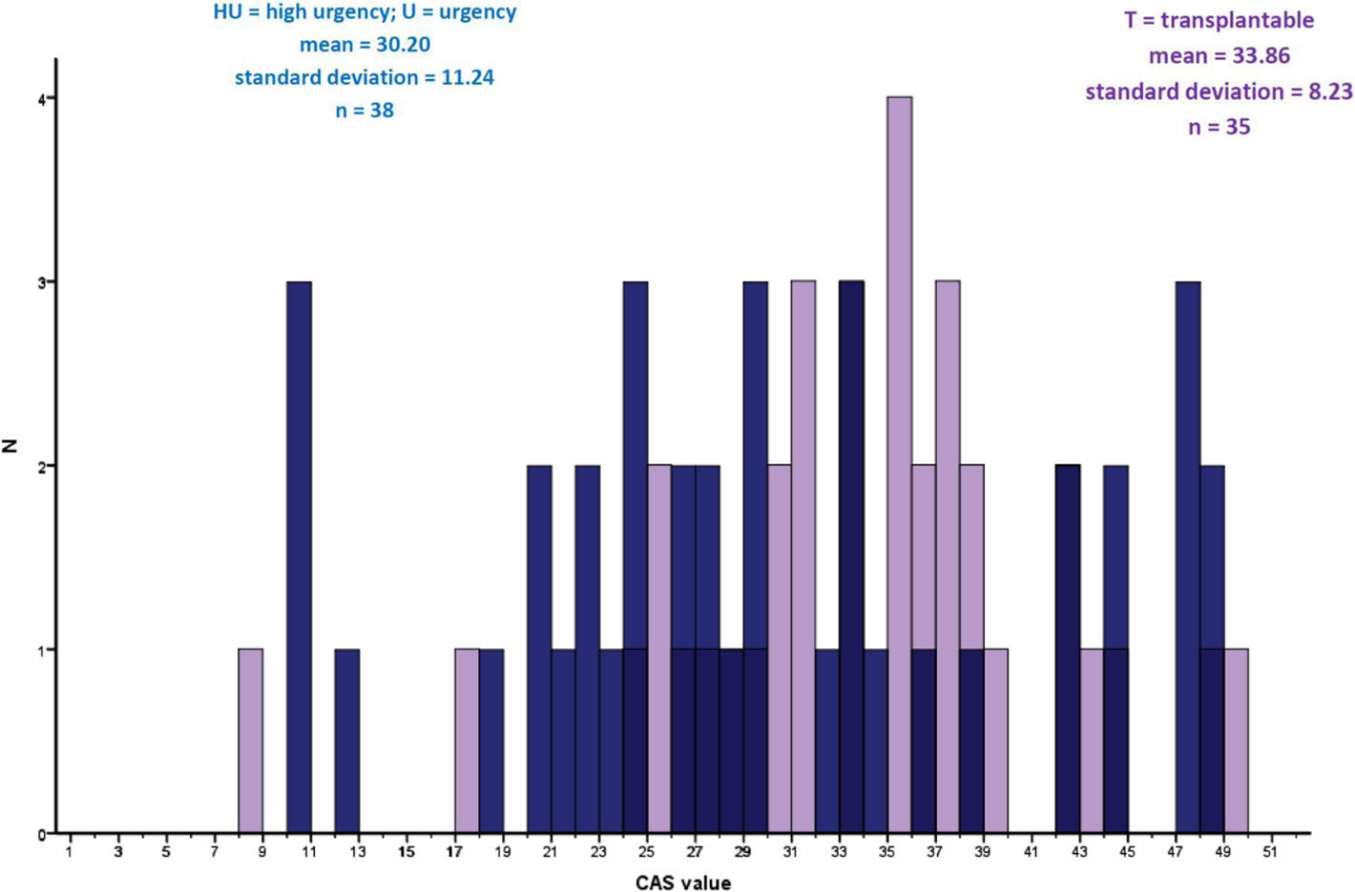
CAS-values of patients transplanted on T status and on HU-status

The SHFM and the impact score were used to calculate a hypothetical retrospective cardiac allocation score for each patient. The distribution of the resulting values with mean of 32 (30–34; 95% confidence interval) is shown in Fig. 4. Patients transplanted from a VAD had on average a lower CAS than patients transplanted without a VAD (mean 26.26 vs 35.29, *p* < 0.001, Fig. 5). There was a large overlap in CAS values between patients transplanted from HU/U vs T status, with a median of 29 (27–34; 95% CI) for the HU/U cohort vs 35 (31–37; 95% CI) for the T cohort (Fig. 6).

## Discussion

The contemporary confrontation with the dilemma of a growing waiting list and a sicker patient population demands a fair allocation system for cardiac transplantation with clearly defined criteria. While medical urgency and waiting time currently determine the priority for organ allocation, the current system in Germany already demands that the treating physicians take into account the expected outcome after transplantation when listing patients for transplant [10]. Yet, how precisely such a prognostic prediction should be made remains undefined and currently leaves ample room for interpretation. In Germany, an increasing public and political interest and involvement in the issue of organ allocation could be observed in the past year, e.g. when the suitability of a pediatric patient with congenital heart disease and cerebral deficits after survived cardiac arrest was controversially discussed. The definition of clear medical and ethical standards is therefore essential to ensure a fair and transparent allocation process and to protect patients and physicians from external influence and judicial compromise.

For patients awaiting lung transplantation, a lung allocation score (LAS) was recently introduced in Germany. In contrast to the current practice in heart transplantation, this score prioritizes patients with a high expected mortality on the waiting list, but only if a high benefit from transplantation is also expected and post post-transplant prognosis is favorable. The recently proposed Cardiac Allocation Score largely follows this reasoning, to maximize the effect of each cardiac transplantation and to gain the most benefit per organ.

Essentially, the proposed CAS is a combination of two scores: one to calculate the expected mortality on the waiting list while the patient is waiting for an organ and a second score to calculate the expected prognosis of the individual patient after transplantation. The detailed methodology is described in the original publication by Smits and coworkers [6]. Briefly, after calculating expected mortality before and after transplantation, these risks are weight against a baseline population risk. The CAS is then derived by subtracting these two values and by performing a statistical adjustment to yield values between 1 and 100.

After evaluation of several prediction models, Smits et al. eventually chose the Seattle Heart Failure Model (SHFM) for prediction of waiting list mortality and the Index for Mortality Prediction After Cardiac Transplantation (IMPACT) score for prediction of prognosis after transplantation. In our current report, we now retrospectively tested the predictive value of two models for estimation of post-transplant survival: The IMPACT and the CARRS-score. We found the IMPACT-score superior in its ability to predict prognosis in our patient population, also because of the small number of patients in our cohort in the high risk stratum of the CARRS-score.

While Smits et al. tested their model in a larger Eurotransplant cohort from whom high urgency or urgency status was requested in an 8 months period, our study describes a mixed population of patients transplanted on all levels of urgency including a large number of patients on T-status. Interestingly, there is a significant overlap in CAS-values between the different levels of urgency, indicating that the inclusion of post-transplant benefit in the allocation algorithm will result in a significant alteration of organ distribution between the current urgency groups. This is a more than marginal affect, as in our patient population the average CAS score of the patients on T-status was even higher than of the patients on HU-status that survived to transplant.

One still open question is how to best include the patients on mechanical support into the distribution algorithm. As these patients usually do not die directly from heart failure, their mortality risk is not adequately reflected by the SHFM-value, which would result in an unwanted disadvantage in the CAS allocation algorithm. The currently proposed solution to this dilemma would be an adjustment of the CAS by 2–3 points, to account for increased non-heart failure related mortality risk, e.g. by infection and stroke. However, in our patient population, the average CAS-value of VAD-supported patients remained on average lower than of non-VAD-supported patients. Interestingly, this was primarily not due to the SHFM-component of the CAS, as one could expect, but due to a higher IMPACT-score. Looking at the individual parameters of this prediction model, infection was significantly more common in VAD-supported patients, which resulted in a higher IMPACT and lower CAS-value. As VAD-related infections can result in an upgrade to high-urgency status (and therefore a higher likelihood of transplantation in the past), one can only speculate on the potential implications of this factor upon introduction of the CAS, when the current high-urgency system will be abandoned. In how far the Cardiac Allocation Score has to be further adjusted for patients with VAD-complications remains to be determined on the solid statistical basis of a larger cohort.

Our study has several limitations. Obviously, this is a retrospective single center study on patients recruited and followed up in our center, therefore all statistical analyses are limited by the sample size. In addition, several of our patients had to be excluded, e.g. because of support by a total artificial heart which does not allow CAS-calculation. Our comparison of CAS-values in patients transplanted on T or HU-status is intrinsically also a comparison of different decades, as in the recent past, transplantation from T-status was a rare exception in our center as well as Germany as a whole. Furthermore, our studies focused exclusively on patients that survived to transplantation and therefore allows no conclusion about the urgency-component of the CAS, as patients that died on the waiting list were not included in the analysis.

## Conclusion

Currenty, the CAS is not used for organ allocation in our institution, but remains one of the possible options for the currently ongoing revision process of organ allocation practice in the Eurotransplant region. Our analysis comprises the first published overview of the distribution of CAS-values in an individual transplant center and gives a first expression how the introduction of the CAS could affect organ allocation in the real world.

## Abbreviations

CAS: Cardiac allocation score
HU: High urgent
LAS: Lung allocation score
SHFM: Seattle heart failure model
T: Transplantable
U: Urgent
VAD: Ventricular assist device
